# Structural Imaging Changes and Behavioral Correlates in Patients with Crohn’s Disease in Remission

**DOI:** 10.3389/fnhum.2016.00460

**Published:** 2016-09-16

**Authors:** Veena A. Nair, Poonam Beniwal-Patel, Ifeanyi Mbah, Brittany M. Young, Vivek Prabhakaran, Sumona Saha

**Affiliations:** ^1^Department of Radiology, School of Medicine and Public Health, University of Wisconsin – Madison, MadisonWI, USA; ^2^Division of Gastroenterology and Hepatology, University of Wisconsin – Madison, MadisonWI, USA; ^3^Medical Scientist Training Program, University of Wisconsin – Madison, MadisonWI, USA; ^4^Neuroscience Training Program, University of Wisconsin – Madison, MadisonWI, USA; ^5^Department of Neurology, University of Wisconsin – Madison, MadisonWI, USA; ^6^Department of Psychology and Department of Psychiatry, University of Wisconsin – Madison, MadisonWI, USA

**Keywords:** neuroimaging, cortical thickness, Crohn’s disease, gut-brain axis

## Abstract

**Background:** Crohn’s disease (CD) is a subtype of inflammatory bowel disease caused by immune-mediated inflammation in the gastrointestinal tract. The extent of morphologic brain alterations and their associated cognitive and affective impairments remain poorly characterized.

**Aims**: We used magnetic resonance imaging to identify structural brain differences between patients with Crohn’s disease in remission compared to age-matched healthy controls and evaluated for structural-behavioral correlates.

**Methods:** Nineteen patients and 20 healthy, age-matched controls were recruited in the study. Group differences in brain morphometric measures and correlations between brain measures and performance on a cognitive task, the verbal fluency (VF) task, were examined. Correlations between brain measures and cognitive measures as well as self-reported measures of depression, personality, and affective scales were examined.

**Results**: Patients showed significant cortical thickening in the left superior frontal region compared to controls. Significant group differences were observed in sub-cortical volume measures in both hemispheres. Investigation of brain-behavior correlations revealed significant group differences in the correlation between cortical surface area and VF performance, although behavioral performance was equivalent between the two groups. The left middle temporal surface area was a significant predictor of VF performance with controls showing a significant positive correlation between these measures, and patients showing the opposite effect.

**Conclusion**: Our results indicate key differences in structural brain measures in patients with CD compared to controls. Additionally, correlation between brain measures and behavioral responses suggest there may be a neural basis to the alterations in patients’ cognitive and affective responses.

## Introduction

It is widely postulated that alterations in brain-gut communication underlie the pathophysiology of irritable bowel syndrome (IBS) wherein altered transmission of sensory nerve pathways from the periphery to the brain results in visceral hypersensitivity (Mayer and Tillisch, 2011). Recent studies also suggest that the inflammatory state of Crohn’s disease (CD), one of the main phenotypes of inflammatory bowel disease (IBD), may also be associated with altered sensory processing. In patients with CD, intestinal inflammatory signals may reach the brain via inflammatory cytokines and cause a cascade of neuroplastic events resulting in alterations in the anatomical and functional substrates of the brain impacting cognitive and affective processing. A recent functional magnetic resonance imaging (fMRI) brain study in patients with active CD therapy revealed decreased pain perception in the brain upon neutralization of the pro-inflammatory cytokine tumor necrosis factor alpha (TNFα; Hess et al., 2015). Furthermore, evidence from studies of other chronic pain disorders suggest that persistent pain can impact cognitive function and emotional decision-making due to a heightened sensitivity to the environment and an inadequate ability to modulate one’s cognitive and emotional states (Bushnell et al., 2013). Notable cognitive deficits include those in the area of attention and memory (Dick and Rashiq, 2007) while prominent affective disturbances are seen in the areas of mood, personality, and quality of life (Lydiard, 2001).

Advanced neuroimaging techniques have been used to enhance understanding of the dysfunction which occurs in the brain-gut axis in patients with functional bowel disorders and with IBD. Studies using structural magnetic resonance imaging (MRI) in IBS have shown cortical morphometric changes in brain regions important for the affective and cognitive aspects of pain as well as gray matter intensity changes in the hypothalamus, a key structure in individuals’ response to stress (Blankstein et al., 2010; Seminowicz et al., 2010). More recently, in patients with IBD, brain MRI showed cortical morphometric changes in the temporal, frontal and parietal cortices as well as microstructural brain abnormalities (Bao et al., 2015). Further data is needed, however, to determine the full range of brain changes which occur in patients with IBD, as well as to show functional relevance of brain differences by investigating differences, if any, in behavior. Patients with IBD often report difficulties with memory and high level processing as well as affect and therefore we chose cognitive and affective tests that, based on the literature, could best capture these processes. The present study therefore aimed to elucidate the structural brain imaging characteristics and behavioral correlates in a group of CD patients in clinical remission compared to a group of age and gender-matched healthy control subjects.

We used surface-based neuroimaging analysis techniques to provide cortical thickness and surface area measurements as these have been shown to be sensitive to cortical morphology. Cortical thickness has been identified as a significant marker of brain pathology (Hutton et al., 2009; Winkler et al., 2010). Several factors such as decreased cell size, death of glial cells and astrocytes, and reduced synaptic density may contribute to changes in cortical thickness in patients with chronic pain (May, 2008). Cortical thickness and surface area are two dissociable measures of cortical surface anatomy that map different phylogeny (Rakic et al., 2009). Current evidence suggests that thickness and area reflect different aspects of cortical neuronal migration – thickness primarily depends on the number of neurons migrating along radial glial fibers within a cortical column, whereas, surface area reflects the number of columns in a cortical region. Furthermore, thickness and surface area have distinguishable genetic underpinnings (Monuki and Walsh, 2001; Chen et al., 2013). A better understanding of the contributions of cortical thickness and surface area will enhance our understanding of the neurobiological mechanisms underlying altered brain morphology with disease (Raznahan et al., 2011; Lin et al., 2014; Wierenga et al., 2014). We, therefore, investigated cortical thickness, surface area, and sub-cortical volume measures and correlation between these measures and cognitive and affective characteristics in patients with CD and matched controls. We chose the letter (phonemic or verbal) fluency task as our main cognitive task, which is extensively used in both clinical and non-clinical populations on account of its ease of administration, face validity (Sauzéon et al., 2011), assessment of both verbal cognitive ability and executive control (Fisk and Sharp, 2004; Federmeier et al., 2010), and high correlation with measures of attention, verbal memory, and word knowledge (Ruff et al., 1997). We used well-validated measures of personality and affect and measures of memory span (digit span), and investigated the relation between these variables and brain measures in both groups. Our hypotheses were: (1) patients with CD in clinical remission will show significant differences in structural brain morphometric measures (cortical thickness, surface area, and sub-cortical volumes) compared to age and gender-matched healthy controls and (2) in patients with CD, brain morphometric measures will significantly correlate with behavioral measures and with measures of pain severity and disease duration.

## Materials and Methods

### Participants

#### Cases

Twenty patients with CD in remission were recruited for this cross-sectional study (one subject was later dropped from the freeSurfer analysis resulting in a final *N* = 19). Eligible patients were 18 years of age or older with a diagnosis of CD as determined by endoscopy, histology, or radiographic imaging. Only subjects in clinical remission defined by a Harvey-Bradshaw Index (HBI) score of less than five were recruited. Patients were excluded if they had a neurological or psychiatric disorder that might affect performance on the task battery we employed in this study, insufficient fluency in English, active or history of substance or alcohol abuse and/or dependence within the past 6 months, contraindications to MRI, or were pregnant or breastfeeding. To eliminate confounds from co-morbidities, patients with other chronic pain disorders (e.g., fibromyalgia, rheumatoid arthritis, irritable bowel syndrome) were excluded. Additionally, patients taking scheduled pain medications (e.g., acetaminophen, non-steroidal anti-inflammatory drugs, narcotics) were also excluded.

#### Controls

Twenty healthy controls, free of any neurological or psychiatric disorders, and matched for age and gender were included in this study. All healthy controls were screened using a detailed self-reported health questionnaire. All controls were medication free and reported no ailments.

All participants provided written informed consent for the study. Subjects received $50 for their participation. The project protocol was reviewed and approved (#H2014-0131) by the health sciences institutional review board at the University of Wisconsin School of Medicine and Public Health.

### Clinical Assessment

Subject demographics including gender, age, education level, and handedness were recorded. CD characteristics including disease location and behavior according to Montreal classification (Satsangi et al., 2006), disease duration, and current medication use were obtained by patient interview and/or review of the medical record.

Harvey-Bradshaw Index was assessed during patients’ routine gastroenterology clinic visits by their usual clinical provider. Study visit for the MRI and behavioral testing was scheduled within 4 weeks of their last clinic visit. Patients were asked to notify the research team if there had been any change in their clinical status between recruitment and their study visit.

Pain intensity was assessed with a 7-day pain diary. Patients were asked to record their pain level daily in the 1 week preceding their study visit using a visual analog scale (VAS; Huskisson, 1974; Bijur et al., 2001; Gallagher et al., 2001, 2002), anchored by “no pain” (score of 0) to “worst possible, unbearable, excruciating pain” (score of 10).

### Neuropsychological Assessment

All participants completed a series of behavioral tasks (cognitive) and questionnaires (affective) outside the scanner during their visit. Tasks performed outside the scanner included the verbal fluency (VF) task, and the digit span forward and backward tasks (Wechsler, 1997). The VF task outside the scanner was administered to all patients by forms of the Controlled Oral Word Association Test (COWAT; Ruff et al., 1996; Sumerall et al., 1997), which requires subjects to produce words beginning with the letters, ‘F,’ ‘A,’ and ‘S’ in three respective 1-min trials. Responses to each letter were recorded and VF scores were based on the total number of correct responses produced by the participants across the three letter conditions. Analyses were done using normed scores corrected for age and education.

In the digit span memory task, increasingly longer strings of numbers were read aloud to the participants. For the forward digit span, subjects were instructed to repeat the numbers in the same order in which these were read to them. For the backward digit span, subjects were instructed to repeat the numbers in an order reverse to what they were read. Total span measure equaling the sum of the scores on the forward and backward spans was calculated for all subjects (Wechsler, 1997).

Affective measures administered included the trait Behavioral Inhibition/Behavioral Activation Scales (BIS/BAS; Carver and White, 1994). The BAS has three subscales: the BAS-Drive subscale measures persistent pursuit of goals (e.g., “When I want something I usually go all-out to get it”); the BAS-Fun Seeking subscale measures desire for new reward (e.g., “I m always willing to try something new if I think it will be fun”); and the BAS-Reward Responsiveness subscale relates to positive responses to reward (e.g., “When I’m doing well at something, I love to keep at it”). The BIS scale taps sensitivity to negative events (e.g., “I feel pretty worried or upset when I think or know somebody is angry at me”).

Mood at the time of the study was assessed using the Positive Activation/Negative Activation Schedules (PANAS; Watson, 1988; Watson et al., 1988a,b). In the state form of the PANAS, participants rated how they “currently feel right now” to each of twenty emotion terms grouped into positive affect ([PA], e.g., elated, happy) and negative affect ([NA], e.g., troubled, upset).

Personality factors were assessed with the 50-item International Personality Item Pool (Goldberg, 1999a,b; Socha et al., 2010). Five personality factors assessed were extraversion, agreeableness, conscientiousness, openness, and emotional stability. Items include different phrases describing people’s behaviors (e.g., “I am interested in people” or “I feel little concern for others”) and participants were instructed to indicate the extent to which they agreed with each statement using a five-point Likert scale where 1 = very inaccurate and 5 = very accurate.

Depression was assessed with the 20-item Center for Epidemiologic studies Depression (CES-D) scale (Devins et al., 1988; Sheehan et al., 1995).

### MRI Acquisition

Magnetic resonance scans were performed on 3 Tesla GE 750 scanners. T1-weighted axial anatomical scans were acquired using FSPGR BRAVO sequence (TR = 8.132 ms, TE = 3.18 ms, TI = 450 ms) over a 256 × 256 matrix and 156 slices (flip angle = 12°, FOV = 25.6 cm, slice thickness = 1 mm).

### MRI Processing

T1-weighted MR images were used for cortical reconstruction and volumetric segmentation and processed with the FreeSurfer image analysis suite (Massachusetts General Hospital, Harvard Medical School^1^) version freeSurfer-Linux-centos6_x86_64-stable-pub-v5.3.0) on a Linux 3.5.0-54-Ubuntu x86_64 machine with Intel(R) Xeon(R) CPU E5-2697 v2 @ 2.70GHz, 24 CPUs. The technical details of FreeSurfer processing, in brief, include motion correction and averaging, removal of non-brain tissue (Segonne et al., 2004), automated Talairach transformation (Talairach and Tournoux, 1988), segmentation of the subcortical white matter and deep gray matter volumetric structures (Fischl et al., 2004; e.g., caudate, putamen, thalamus), intensity normalization, tessellation of the gray matter and white matter boundary, automated topology correction (Fischl et al., 2001), and surface deformation following intensity gradients to effectively place the boundaries between brain tissue (CSF, WM, and GM). FreeSurfer calculates thickness as the closest distance from the gray matter–white matter boundary to the gray matter–CSF boundary at each vertex on the tessellated surface (Fischl and Dale, 2000). Before group analysis all data were visually checked for data quality and registration errors. One subject’s data was dropped because of poor scan quality.

### Statistical Analysis

All measures were examined for normality using the Shapiro–Wilk test. Between-group differences in demographic, clinical, affective, and cognitive variables were assessed by using the Mann–Whitney *U*-test for (non-normal) continuous variables and the Fisher exact test for categorical variables. For the variables with a normal distribution we applied the multivariate ANOVA to identify group differences.

We performed the following analyses:

(1) *Behavioral differences:* parametric (or non-parametric, if relevant) analyses were performed to examine group differences on behavioral measures of VF, memory span, affect, and personality factors.
(2) *Brain-based measures – Cortical level*: a vertex-by-vertex analysis was used to assess differences in cortical thickness and cortical surface area between control subjects and patients. These analyses were undertaken using FreeSurfer’s statistical tool Qdec. Cortical thickness and cortical surface area were modeled as a function of group, controlling for age, sex, and whole-hemisphere average cortical thickness, and cortical surface area, respectively. Data were smoothed with a 15-mm full-width half-maximum (FWHM) to improve inter-subject variability. To correct for multiple comparisons, a Qdec Monte Carlo simulation was implemented with cluster-forming threshold set to *p* < 0.05.
(3) *Brain and behavior – Cortical level*: the association between cortical thickness and surface area and performance on the VF task was investigated using Qdec. Age, sex, whole-hemisphere average cortical thickness (or average cortical surface area) were included in these analyses as covariates.
(4) *Brain-based measures – Sub-cortical level*: sub-cortical volume measures were normalized to a z-score before evaluating between-group differences using a general linear model (MANCOVA) for each hemisphere with age, sex, and intracranial volume as covariates and group status (patient vs. control) as the independent factor. For the purposes of correction for multiple comparisons, *p* = 0.008 (= 0.05/6 regions in each hemisphere – thalamus, caudate, putamen, pallidum, hippocampus, and the amygdala) was considered significant.
(5) *Brain and behavior – Sub-cortical level*: correlation analyses between sub-cortical volume measurements and clinical, affective, and cognitive variables after controlling for intracranial volume were performed. Correlation results are reported at unadjusted *p* < 0.05 given the limited sample size.

## Results

### Demographic Characteristics and Clinical Features

Details regarding demographic and clinical characteristics of the study participants are provided in **Table 1**. There were no significant group differences on age (*p* = 0.84), sex (*p* = 0.74), education (*p* = 0.30), or handedness (*p* = 0.60).

**Table 1:** Demographic and clinical characteristics of study participants.

Characteristic	Healthy Controls	Patients with CD	*p*-value
No. of subjects (N)	20	20	
No. of males (N)	14	12	0.74
Mean age (SD)	37.0 16.23)	35.8 (15.81)	0.84
Handedness (right/total)	(17/19)	(16/19)	0.60
Mean years of education (SD)	16.7 (1.9)	16.02 (2.6)	0.30
Median disease duration in years (SD)	N/A	11.4 (9.0)	
Disease location (N)	N/A	L1 5 L2 3 L3 11 L4 3	
Disease behavior (N)	N/A	B1 11 B2 6 B3 0 P 2	
IBD medications	N/A	Antibiotics 0 5-ASA 7 Immunomodulator 6 Anti-TNFα 9 Anti-Integrin 1 Corticosteroids 0	
Average pain score (SD) (VAS, 0-10)	N/A	0.79 (0.84)	
Mean depression score (SD)	10.15(6.23)	11.21(6.48)	0.61

*5-ASA, 5 aminosalicylate; anti-TNFα, anti-tumor necrosis factor alpha agent; VAS, visual analog scale.*

### Crohn’s Disease Characteristics

All patients were in clinical remission and taking at least one CD maintenance medication (see **Table 1**). The majority of patients had ileocolonic distribution of CD (55%), while 25% had isolated ileal involvement and 15% had isolated colonic disease. Fifteen percent of patients had perianal disease. Most patients had non-stricturing, non-penetrating CD (55%).

#### Behavioral Measures

In the control group, the variables of depression, PANAS negative affect, personality factor of Emotional Stability, and in the patient group, depression and pain scores, and the digit span forward score were non-normal (*p* < 0.05). Reported *p*-values are therefore based on the non-parametric Mann–Whitney *U*-test for continuous variables. **Table 2** indicates the group differences on these variables.

**Table 2:** Group differences in affective and cognitive measures.

Variable	Healthy controls (*N* = 20)	Patients with IBD (*N* = 19)	*p*-value
BAS reward responsiveness	16.50 (2.33)	17.00 (1.59)	0.72
BAS fun seeking	12.60 (2.52)	15.36 (1.46)	0.00^∗^
BAS drive	11.10 (2.15)	27.36 (2.43)	0.00^∗^
BIS	21.15 (3.30)	25.58 (2.11)	0.00^∗^
Positive affect	32.70 (9.14)	27.21 (8.18)	0.11
Negative affect	12.80 (3.20)	13.63 (3.15)	0.34
Extraversion	29.55 (2.72)	30.16 (2.52)	0.39
Agreeableness	30.50 (2.58)	29.84 (3.55)	0.44
Conscientiousness	31.50 (3.00)	32.63 (4.09)	0.31
Emotional stability	25.65 (5.50)	27.84 (4.14)	0.13
Openness	33.35 (2.34)	31.68 (3.19)	0.09^a^
Digit span forward	11.75 (2.55)	12.36 (8.74)	0.17
Digit span backward	9.10 (2.88)	7.05 (1.84)	0.01^∗^
Digit span total	20.85 (4.99)	17.68 (3.32)	0.02^∗^
Verbal fluency raw	45.55 (10.82)	43.79 (12.22)	0.55
Verbal fluency normed	0.13 (0.98)	-0.03 (1.04)	0.63

*BIS, Behavioral Inhibition Scale; BAS, Behavioral Approach Scale; ^∗^*p* < 0.05; ^a^0.05 < *p* < 0.10.*

#### Brain Based Measures – Cortical Level

##### Cortical thickness analysis

###### Left hemisphere

**FIGURE 1:**
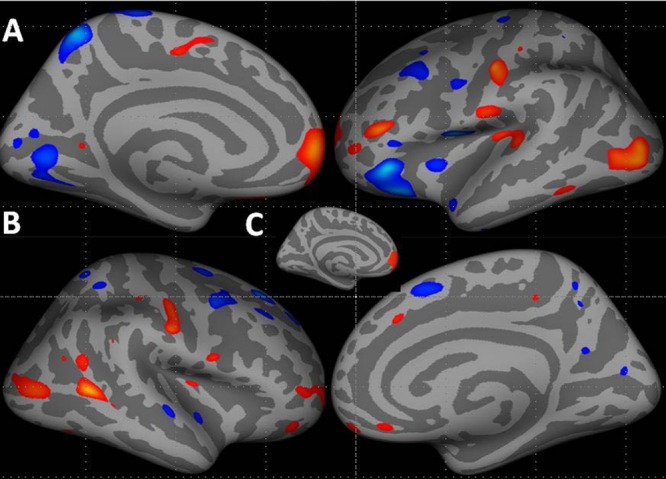
**Increase in cortical thickness (warm colors) in the left hemisphere (**A** – medial and lateral surfaces) and right hemisphere (**B** – lateral and medial surfaces) in patients compared to healthy controls.** All vertices with threshold between 0.01 < *p* < 0.0001, uncorrected, shown for display purposes. **(C)** Inset shows the cluster in the left hemisphere superior frontal region that survived correction for multiple comparisons, *p* < 0.05. Blue–cyan represents cortical thinning; red represents cortical thickening.

Patients showed an increased average left hemisphere cortical thickness (mean, 2.68 mm ± 0.17 [standard deviation]) compared with control subjects (mean, 2.66 mm ± 0.12, *p* < 0.01, corrected; **Figure 1A**; Supplementary Table S1 summarize the results of vertex-by-vertex comparison of cortical thickness between patients and healthy control subjects). Compared with control subjects, patients showed increased thickness in the left superior frontal region that survived correction for multiple comparisons (**Figure 1C**). Other regions showing increased thickness in patients compared to controls included the lateral occipital, post-central, and the superior and inferior temporal regions.

###### Right hemisphere

Although, several regions in the right hemisphere showed group differences in cortical thickness, none of these survived correction for multiple comparisons. Compared with control subjects, patients showed increased thickness in the right middle temporal, post-central, lateral occipital, and inferior parietal regions. Compared with controls, patients showed cortical thinning in several middle and superior frontal, superior temporal, superior parietal, precuneus, and pericalcarine regions (**Figure 1B**; Supplementary Table S1).

##### Cortical surface area analysis

**FIGURE 2:**
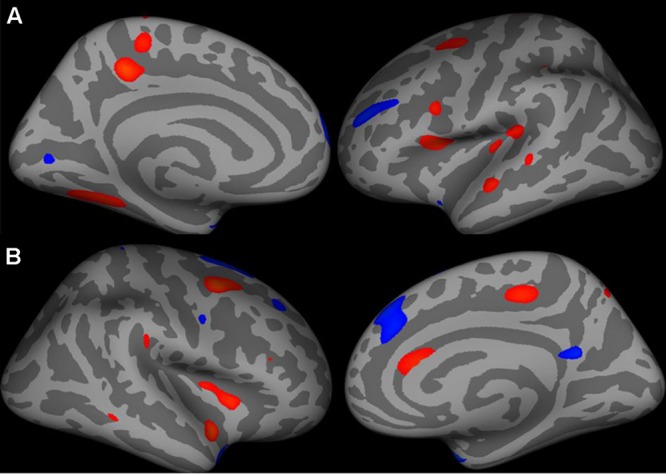
**Increase in cortical surface area (warm colors) in the left hemisphere (**A** – medial and lateral surfaces) and right hemisphere (**B** – lateral and medial surfaces) in patients compared to healthy controls.** All vertices with threshold between 0.01 < *p* < 0.0001, uncorrected, shown for display purposes.

Supplementary Table S2 provides detailed results of vertex-by-vertex comparison of cortical surface area between CD patients and healthy controls (see also **Figure 2**).

###### Left hemisphere

Average left cortical surface area showed a trend toward significant decrease between patients (mean, 2627.9 mm^2^ ± 277.68) and controls (mean, 2779.4 mm^2^ ± 269.65) (*p* = 0.09, uncorrected). Compared with controls, patients had decreased surface area (although this difference did not survive corrections for multiple testing) in several regions including the left precuneus, pars opercularis, fusiform, paracentral, superior temporal and caudal middle frontal and insular regions. Compared with controls, patients had increased surface area in several regions including the temporal pole, superior frontal regions (although this difference did not survive corrections for multiple testing).

###### Right hemisphere

Average right cortical surface area did not differ between patients (mean, 1203.59 mm^2^ ± 130.5) and controls (mean, 1272.32 mm^2^ ± 118.35) (*p* = 0.11, uncorrected). Compared with control subjects, patients had decreased surface area in several regions including the right caudal middle frontal, superior temporal, insula, paracentral, and caudal anterior cingulate regions. Compared with control subjects, patients had increased surface area in several regions including the temporal pole, superior frontal, precuneus, and precentral regions.

#### Brain-Behavior Correlations – Cortical Level

##### Cortical measures and verbal fluency task performance

**FIGURE 3:**
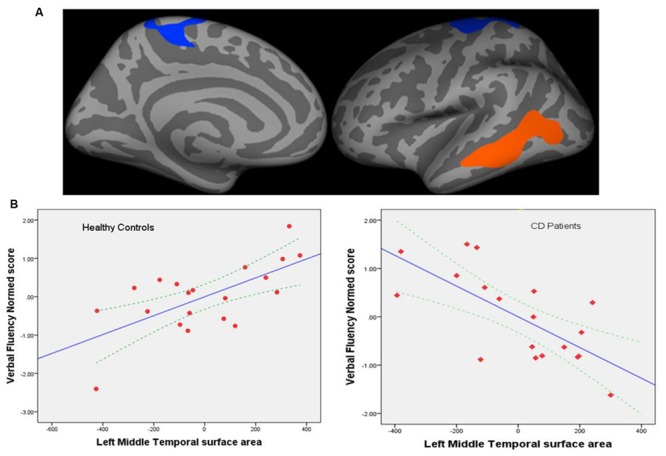
**(A)** Regional cortical surface areas showing significant group differences in the correlation between the left cortical surface area and normed verbal fluency scores. Left precentral (*p* = 0.008) and middle temporal regions (*p* = 0.002) showed significant effects (corrected for multiple comparisons). Blue-cyan represents negative association; red represents positive association. **(B)** Graphs showing correlation between cortical surface area (mm^2^) for the left middle temporal region and verbal fluency performance for controls (left) and patients (right). Dotted lines represent 95% confidence interval (CI) for the mean. The left middle temporal surface area was the most significant predictor of performance with standardized coefficient β = 0.90, *p* = 0.005; left precentral area only showed a trend toward significance with β = -0.496, *p* = 0.09 and is therefore not shown here.

There were no significant group differences in correlations between cortical thickness and performance on the VF task. FreeSurfer analyses with Qdec showed significant group differences in the correlation between the left cortical surface area and normed VF scores. Specifically clusters in the left middle temporal regions (*p* = 0.002) and the precentral region (*p =* 0.008) showed significant effects (corrected for multiple comparisons; **Figure 3A**).

Hierarchical multiple regression (HMR) models were run in SPSS for each group to further elucidate these results (**Figure 3B**).

###### Patients

In the first step of the HMR, three predictors of age, sex, and left hemisphere mean surface area were entered. This model was not statistically significant *F*(3,15) = 1.49, *p* = 0.26. After the entry of the left middle temporal area and precentral area as predictors at step 2, the model was statistically significant *F*(5,13) = 4.64, *p* = 0.012, and total variance explained by the model as a whole was 64%. The surface area for these regions explained an additional 42% of the variance (*R* square change = 0.42, *p* < 0.007). The left middle temporal surface area was the most significant predictor of performance with standardized coefficient β = -1.22, *p* = 0.005 (**Figure 3B**, right panel); left precentral area was not a significant predictor with β = 0.44, *p* = 0.27.

###### Controls

A similar regression model as above was tested. The regression model with age, sex, and left hemisphere mean surface area as covariates and regional surface area in the left middle temporal and precentral regions was significant [*F*(5,14) = 4.597, *p* < 0.011]. The left middle temporal and precentral surface areas explained an additional 45% of the variance (*R* square change = 0.447, *p* < 0.004). The left middle temporal surface area was the most significant predictor of performance with standardized coefficient β = 0.90, *p* = 0.005 (**Figure 3B**, left panel); only the left precentral area showed a trend toward significance with β = -0.496, *p* = 0.09.

##### Cortical measures and other behavioral performance and clinical measures

Measures of regional cortical thickness or surface area as predictors failed to show any significant association with the other behavioral measures, or disease duration, or pain.

#### Brain-Based Measures – Sub-cortical Volumes

We investigated between-group analysis in sub-cortical volumes with a general linear model (MANCOVA) for each hemisphere with age and sex as covariates, group status (patient vs. control) as the independent factor, and sub-cortical volumes in each hemisphere (thalamus, caudate, putamen, pallidum, hippocampus, and amygdala) as the dependent variables. Overall *F*-test indicated significant group differences between patients and controls in the left hemisphere sub-cortical volumes [*F*(6,29) = 3.37, *p* = 0.012, η_p_^2^ = 0.41]. In specific sub-cortical volumes, there were trends toward significance, in the left caudate, *p* = 0.09, left putamen, *p* = 0.03, and the left pallidum, *p* = 0.01, uncorrected. Patients showed reduced sub-cortical volumes compared to controls. Overall *F*-test also indicated significant group differences between patients and controls in the right hemisphere sub-cortical volumes [*F*(6,29) = 3.16, *p* = 0.01, η_p_^2^ = 0.39], with trends toward significant differences in the right putamen, *p* = 0.01, and the right pallidum, *p* = 0.03, uncorrected (see **Table 3** for sub-cortical volumes measures in the two groups).

**Table 3:** Sub-cortical volume measures in patients and controls.

Region	Patients (*N* = 19)	Controls (*N* = 20)
Left thalamus	7891.96 (1024.47)	8294.87 (1112.14)
Left caudate	3938.5 (603.16)	3926.37 (571.65)
^+^Left putamen	5560.68 (737.04)	6248.6 (1076.26)
^∗^Left pallidum	1478.23 (247.89)	1745.75 (325.96)
Left hippocampus	4198.8 (427.03)	4284.19 (465.35)
Left amygdala	1578.98 (189.40)	1694.30 (232.19)
Right thalamus	7225.18 (875.95)	7334.22 (781.66)
Right caudate	4055.41 (577.13)	4077.22 (627.44)
^∗^Right putamen	5407.43 (584.22)	6023.01 (1021.15)
^∗^Right pallidum	1559.17 (222.40)	1745.41 (231.49)
Right hippocampus	4319.53 (389.26)	4408.915 (500.70)
Right amygdala	1784.59 (146.52)	1837.46 (209.99)

*^∗^*p* < 0.05; ^+^0.05 < *p* < 0.10.*

#### Brain-Behavior Correlations – Sub-cortical Level

##### Sub-cortical volumes and verbal fluency and other behavioral measures

Although, both patient (Supplementary Table S3A) and control (Supplementary Table S3B) groups showed significant correlations, there were fewer correlations in patients. Brain volumes that correlate with certain behavioral measures in patients do not correlate or show the opposite correlation with these measures in healthy controls and vice versa.

## Discussion

There is evidence that patients with IBS as well as other chronic pain syndromes such as fibromyalgia, chronic low back pain, and headache/migraine (May, 2008; Blankstein et al., 2010; Seminowicz et al., 2010; Schwedt et al., 2015) have structural brain abnormalities including regional cortical thickening and thinning and gray matter density changes compared to healthy individuals. Similarly, patients with chronic inflammatory conditions such as chronic pancreatitis and rheumatoid arthritis demonstrate structural changes including reduced cortical thickness in the brain areas involved with pain processing and altered subcortical gray matter content in the basal ganglia, respectively (Frokjaer et al., 2012; Wartolowska et al., 2012). It has been suggested that the loss of brain gray matter in patients suffering from chronic pain may represent the neuroanatomical substrate for pain memory and that given the overlap in the brain changes seen among the various patient populations, chronic pain patients may have a common “brain signature” in areas known to be involved in pain regulation (May, 2008).

In the present study we characterized the structural differences in a group of patients with CD in clinical remission compared to healthy controls. Studies from Graff et al. (2006) have shown that CD patients in remission report a compromised quality of life compared to the general population while pain anxiety and pain-specific catastrophizing are not associated with disease activity. Better understanding of brain morphometry in these patients may explain these perplexing findings. We hypothesized that patients with CD would show significant morphometric changes possibly due to alterations in the brain network involved in detecting and orienting attention to salient sensory events. Our study provides evidence that compared to age and gender-matched healthy controls, patients with CD show morphometric brain differences involving regional cortical thickening and differences in sub-cortical volumes.

### Patients Show Significant Differences in Structural Brain Measures Compared to Age and Gender-Matched Controls

We found that patients with CD had cortical thickening in the left superior frontal region compared to controls. This is consistent with the area recently reported by Liu et al. (2016) to show significantly increased thickness in women with primary dysmenorrhea. Notably, the superior medial frontal region (Brodmann Area 10) identified in this study is a key area implicated in the control and monitoring of action, specifically in monitoring the reward value of ongoing cognitive processes (Pochon et al., 2002). It is also engaged in affective processing and in the monitoring of outcomes associated with punishment or reward (Amodio and Frith, 2006), in working memory processes and executive functions (Christoff and Gabrieli, 2000), as well as in the putative pain matrix. This result suggests that IBD patients, although currently in remission, may suffer from the long term effects of chronic discomfort and pain which may lead to brain changes not only in the pain network but also in the cognitive and affective networks that modulate pain. Cortical thickening is influenced by factors such as the extent of myelination, alterations in cell size, dendritic spines or synaptic density (Sowell et al., 2003) as well as changes in interactions within neuronal circuits that are predominantly inhibitory or excitatory (Cesa et al., 2007). There is evidence that these changes are occurring continuously through maturation and aging (Terry et al., 1987; Benes et al., 1994; Huttenlocher and Dabholkar, 1997), as well as with injury (Ito et al., 2006) or drug treatments (Li et al., 2007). It is likely that changes in cortical thickness in patients compared to controls may be the result of a confluence of factors such as the prolonged exposure to pain that may drive changes in the excitatory (e.g., NMDA-ergic) or inhibitory (e.g., GABA-ergic) systems as well as responses to various medications.

Cortical thickening in the left superior frontal region parallels the group differences seen in measures of behavioral inhibition (suggesting greater sensitivity to external stimuli), behavioral activation sub-scales (more fun-seeking, more persistence in seeking goals) as well as the observed differences on the memory span tasks between CD patients and controls.

Although changes in cortical thickness were noted in several other regions including post-central, para-central, orbital and middle frontal, and cingulate regions, these did not survive correction for multiple comparisons. We postulate that our small sample size and the wide variability in the disease location, behavior, and duration among our CD patients in this study may have impeded our ability to detect changes in more regions at the group level.

In contrast to the often reported cortical changes in the insular and cingulate regions (key areas in pain processing) in other conditions associated with chronic pain (e.g., IBS, migraine; Davis et al., 2008; Schwedt et al., 2015) we did not find changes in these regions in our CD cohort that survived testing for multiple comparisons. These findings of regional structural changes may be due to the higher pain levels reported by patients in those studies, compared to patients in our study wherein the average pain reported in the week before participation ranged from 0 to 2 suggesting very mild to no pain.

We found differences in *cortical surface area* between CD patients and controls; however, these did not survive correction for multiple comparisons. Regions that showed differences in surface area included, the left and right superior and middle frontal regions, bilateral temporal poles, as well as the right insular region. In addition to the well-established roles for the superior and middle frontal regions in cognitive control and responding to behaviorally salient events, the other regions noted in this analysis such as the bilateral temporal pole and insular regions have been implicated in socioemotional processing (i.e., coordinating awareness of body feelings, contextual social cues, emotionally salient stimuli, etc.). The temporal poles are also known to provide information regarding stimulus context or salience to the insula (Olson et al., 2007; Craig, 2009). This suggests that pain driven morphological changes in these regions could influence the manner in which CD patients process their external environment.

With respect to *sub-cortical volume* measures (**Table 3**), CD patients overall showed reduced volumes compared to controls with the exception of the left caudate, where controls showed slightly reduced volume compared to patients. Several morphometric studies have reported decreases in gray matter volumes in patients with chronic pain disorders such as migraine, fibromyalgia (Kuchinad et al., 2007), chronic tension headache (Schmidt-Wilcke et al., 2005), chronic low back pain (Mao and Yang, 2015), and IBS (Davis et al., 2008). Similar to our findings Davis et al reported a reduction in thalamic volume in IBS patients compared to controls. Reduced volumes may be due to cell atrophy, neural or glial cell apoptosis, or synaptic loss and may also be influenced by factors such as disease duration and pain severity (May, 2008). We also posit that medications or other disease-related treatments may have affected the gray matter volume changes we observed in our CD cohort.

### In the Patient Group, Cortical and Sub-cortical Measures Correlate with Behavioral Measures

#### Cortical Measures and Behavioral Correlations

With regards to our brain-behavioral correlations, we found significant associations between the left middle temporal cortical surface area and performance on the VF task in both patients and controls, albeit in different directions. Given the behavioral equivalence in performance on the VF task outside the scanner between the two groups but brain based differences in cortical surface area, our findings suggest that brain based biomarkers may be more sensitive (than behavior) to the effects of dysregulation of the brain-gut axis and subsequent alterations in behavior in the long run. It is also likely that patients may engage in compensatory mechanisms to produce the same behavioral output as matched controls, a trend that has been noted with increasing age and is best investigated through functional imaging studies (Rypma and D’Esposito, 2000; Goh and Park, 2009). Results of a pilot fMRI study from our group (Beniwal-Patel et al., 2016) suggests that patients may engage in compensatory mechanisms to maintain behavioral performance (for e.g., in healthy control subjects the left hemisphere is mainly involved in processing language but in patients, corresponding areas in the right hemisphere may also be involved).

#### Subcortical Measures and Behavioral Correlations

We also found that both pain and disease duration negatively correlated with subcortical volume measures. This suggests that greater pain and longer duration of disease correlate with smaller volumes of the left putamen and amygdala and the right caudate and hippocampus. Both structural and functional brain imaging studies in pain processing indicate decreased gray matter and reduced functional activation in the hippocampus and amygdala in response to painful stimuli, and in the basal ganglia (which is comprised of both the caudate and putamen) in response to withdrawal from pain (Bingel et al., 2002; Rodriguez-Raecke et al., 2009) However, we should note that these brain-behavior correlations are reported at liberal statistical threshold uncorrected for multiple comparisons and should therefore be considered as an exploratory analysis. We did not observe any significant correlations between subcortical volumes and other affective and personality measures on which patients differed significantly from controls (i.e., the BAS fun seeking, drive, BIS, and the memory span measures).

### Limitations

Our study was limited by small sample size. However, given that our CD patients were closely matched to healthy control subjects in age, gender, handedness, and education, we believe that the findings reported here with respect to brain morphological changes merit further investigation with larger sample sizes to more clearly elucidate some of the findings that failed to reach statistical significance after correcting for multiple comparisons. Another limitation in our study was the failure to control for any differences in IQ which is known to impact surface based morphometric analysis. We did collect information regarding the number of years of education and found no significant group differences on education. Several studies have reported a correlation of ∼0.5 between IQ and education when measured at the same time (Johnson et al., 2005; Batty et al., 2007; Deary and Johnson, 2010). Given that these two variables show moderate to high correlations and that there were no significant group differences on education we expect our results to hold even if IQ were used as a covariate. Future studies examining CD patients based on medication use, disease phenotype, duration of disease and cognitive ability as measured by IQ are needed as these factors may be implicated in the brain changes observed in our group of patients.

## Conclusion

We found differences in structural brain measures between patients with CD and healthy, age-matched controls. Specifically, patients with CD demonstrated cortical thickening in the left superior frontal cortex and decreased sub-cortical volumes with few exceptions. Additionally, we found correlation or trends for correlations between brain measures and behavioral responses suggesting that structural brain changes associated with CD may lead to alterations in the way that CD patients process and respond to emotional and painful stimuli. Future neuroimaging studies evaluating the impact of factors such as disease duration, severity, and treatment on these structural abnormalities are needed to better understand the brain-gut relationship in CD.

## Author Contributions

VP, SS conceived of the study; VN, IM, BY helped with data collection; VN performed neuroimaging and behavioral data analysis aided by IM; PB-P and SS compiled the clinical information; VN wrote the manuscript with inputs from VP and SS; PB-P, IM, BY provided comments on the manuscript; VN, VP, SS discussed the results and implications of the study.

## Conflict of Interest Statement

Dr. SS is a consultant for UCB Biosciences, Inc. All the other authors declare that the research was conducted in the absence of any commercial or financial relationships that could be construed as a potential conflict of interest.

The reviewer FH and handling Editor declared their shared affiliation, and the handling Editor states that the process nevertheless met the standards of a fair and objective review.

## Footnotes

1 http://surfer.nmr.mgh.harvard.edu/

## References

[B1] Amodio D. M. Frith C. D. (2006). Meeting of minds: the medial frontal cortex and social cognition. *Nat. Rev. Neurosci.* 7 268–277. 10.1038/nrn1884 16552413

[B2] Bao C. H. Liu P. Liu H. R. Wu L. Y. Shi Y. Chen W. F. (2015). Alterations in brain grey matter structures in patients with crohn’s disease and their correlation with psychological distress. *J. Crohns Colitis* 9 532–540. 10.1093/ecco-jcc/jjv057 25895879

[B3] Batty G. D. Deary I. J. Macintyre S. (2007). Childhood IQ in relation to risk factors for premature mortality in middle-aged persons: the Aberdeen Children of the 1950s study. *J. Epidemiol. Community Health* 61 241–247. 10.1136/jech.2006.048215 17325403 PMC2652919

[B4] Benes F. M. Turtle M. Khan Y. Farol P. (1994). Myelination of a key relay zone in the hippocampal formation occurs in the human brain during childhood, adolescence, and adulthood. *Arch. Gen. Psychiatry* 51 477–484. 10.1001/archpsyc.1994.03950060041004 8192550

[B5] Beniwal-Patel P. Nair V. A. Prabhakaran V. Saha S. (2016). Sa1886 altered brain functional activation and connectivity patterns in patients with Crohn’s disease in remission. *Gastroenterology* 150:S392 10.1016/s0016-5085(16)31375.0

[B6] Bijur P. E. Silver W. Gallagher E. J. (2001). Reliability of the visual analog scale for measurement of acute pain. *Acad. Emerg. Med.* 8 1153–1157. 10.1111/j.1553-2712.2001.tb01132.x 11733293

[B7] Bingel U. Quante M. Knab R. Bromm B. Weiller C. Büchel C. (2002). Subcortical structures involved in pain processing: evidence from single-trial fMRI. *Pain* 99 313–321. 10.1016/S0304-3959(02)00157-4 12237210

[B8] Blankstein U. Chen J. Diamant N. E. Davis K. D. (2010). Altered brain structure in irritable bowel syndrome: potential contributions of pre-existing and disease-driven factors. *Gastroenterology* 138 1783–1789. 10.1053/j.gastro.2009.12.043 20045701

[B9] Bushnell M. C. Čeko M. Low L. A. (2013). Cognitive and emotional control of pain and its disruption in chronic pain. *Nat. Rev. Neurosci.* 14 502–511. 10.1038/nrn3516 23719569 PMC4465351

[B10] Carver C. S. White T. L. (1994). Behavioral-inhibition, behavioral activation, and affective responses to impending reward and punishment – the Bis Bas scales. *J. Pers. Soc. Psychol.* 67 319–333. 10.1037/0022-3514.67.2.319

[B11] Cesa R. Scelfo B. Strata P. (2007). Activity-dependent presynaptic and postsynaptic structural plasticity in the mature cerebellum. *J. Neurosci.* 27 4603–4611. 10.1523/JNEUROSCI.5617-06.2007 17460073 PMC6673001

[B12] Chen C. H. Fiecas M. Gutierrez E. D. Panizzon M. S. Eyler L. T. Vuoksimaa E. (2013). Genetic topography of brain morphology. *Proc. Natl. Acad. Sci. U.S.A.* 110 17089–17094. 10.1073/pnas.1308091110 24082094 PMC3801007

[B13] Christoff K. Gabrieli J. D. (2000). The frontopolar cortex and human cognition: evidence for a rostrocaudal hierarchical organization within the human prefrontal cortex. *Psychobiology* 28 168–186. 10.3758/BF03331976

[B14] Craig A. D. (2009). How do you feel—now? The anterior insula and human awareness. *Nat. Rev. Neurosci.* 10 59–70. 10.1038/nrn2555 19096369

[B15] Davis K. Pope G. Chen J. Kwan C. Crawley A. Diamant N. (2008). Cortical thinning in IBS: implications for homeostatic, attention, and pain processing. *Neurology* 70 153–154. 10.1212/01.wnl.0000295509.30630.10 17959767

[B16] Deary I. J. Johnson W. (2010). Intelligence and education: causal perceptions drive analytic processes and therefore conclusions. *Int. J. Epidemiol.* 39 1362–1369. 10.1093/ije/dyq072 20504860

[B17] Devins G. M. Orme C. M. Costello C. G. Binik Y. M. Frizzell B. Stam H. J. (1988). Measuring depressive symptoms in illness populations: psychometric properties of the center for epidemiologic studies depression (CES-D) scale. *Psychol. Health* 2 139–156. 10.1080/08870448808400349

[B18] Dick B. D. Rashiq S. (2007). Disruption of attention and working memory traces in individuals with chronic pain. *Anesth. Analg.* 104 1223–1229. 10.1213/01.ane.0000263280.49786.f5 17456678

[B19] Federmeier K. D. Kutas M. Schul R. (2010). Age-related and individual differences in the use of prediction during language comprehension. *Brain Lang.* 115 149–161. 10.1016/j.bandl.2010.07.006 20728207 PMC2975864

[B20] Fischl B. Dale A. M. (2000). Measuring the thickness of the human cerebral cortex from magnetic resonance images. *Proc. Natl. Acad. Sci. U.S.A.* 97 11050–11055. 10.1073/pnas.200033797 10984517 PMC27146

[B21] Fischl B. Liu A. Dale A. M. (2001). Automated manifold surgery: constructing geometrically accurate and topologically correct models of the human cerebral cortex. *IEEE Trans. Med. Imaging* 20 70–80. 10.1109/42.906426 11293693

[B22] Fischl B. Van Der Kouwe A. Destrieux C. Halgren E. Segonne F. Salat D. H. (2004). Automatically parcellating the human cerebral cortex. *Cereb. Cortex* 14 11–22. 10.1093/cercor/bhg087 14654453

[B23] Fisk J. E. Sharp C. A. (2004). Age-related impairment in executive functioning: updating, inhibition, shifting, and access. *J. Clin. Exp. Neuropsychol.* 26 874–890. 10.1080/13803390490510680 15742539

[B24] Frokjaer J. B. Bouwense S. A. Olesen S. S. Lundager F. H. Eskildsen S. F. Van Goor H. (2012). Reduced cortical thickness of brain areas involved in pain processing in patients with chronic pancreatitis. *Clin. Gastroenterol. Hepatol.* 10 434–438.e1. 10.1016/j.cgh.2011.11.024 22155560

[B25] Gallagher E. J. Bijur P. E. Latimer C. Silver W. (2002). Reliability and validity of a visual analog scale for acute abdominal pain in the ED. *Am. J. Emerg. Med.* 20 287–290. 10.1053/ajem.2002.33778 12098173

[B26] Gallagher E. J. Liebman M. Bijur P. E. (2001). Prospective validation of clinically important changes in pain severity measured on a visual analog scale. *Ann. Emerg. Med.* 38 633–638. 10.1067/mem.2001.118863 11719741

[B27] Goh J. O. Park D. C. (2009). Neuroplasticity and cognitive aging: the scaffolding theory of aging and cognition. *Restor. Neurol. Neurosci.* 27 391–403. 10.3233/RNN-2009-0493 19847066 PMC3355626

[B28] Goldberg L. R. (1999a). A broad-bandwidth, public domain, personality inventory measuring the lower-level facets of several five-factor models. *Pers. Psychol. Eur.* 7 7–28.

[B29] Goldberg L. R. (1999b). The curious experiences survey, a revised version of the dissociative experiences scale: factor structure, reliability, and relations to demographic and personality variables. *Psychol. Assess.* 11 134–145. 10.1037/1040-3590.11.2.134

[B30] Graff L. A. Walker J. R. Lix L. Clara I. Rawsthorne P. Rogala L. (2006). The relationship of inflammatory bowel disease type and activity to psychological functioning and quality of life. *Clin. Gastroenterol. Hepatol.* 4 1491–1501. 10.1016/j.cgh.2006.09.027 17162241

[B31] Hess A. Roesch J. Saake M. Sergeeva M. Hirschmann S. Neumann H. (2015). Functional brain imaging reveals rapid blockade of abdominal pain response upon anti-TNF therapy in crohn’s disease. *Gastroenterology* 149 864–866. 10.1053/j.gastro.2015.05.063 26264347

[B32] Huskisson E. C. (1974). Measurement of pain. *Lancet* 304 1127–1131. 10.1016/S0140-6736(74)90884-8 4139420

[B33] Huttenlocher P. R. Dabholkar A. S. (1997). Regional differences in synaptogenesis in human cerebral cortex. *J. Comp. Neurol.* 387 167–178. 10.1002/(SICI)1096-9861(19971020)387:2<167::AID-CNE1>3.0.CO;2-Z 9336221

[B34] Hutton C. Draganski B. Ashburner J. Weiskopf N. (2009). A comparison between voxel-based cortical thickness and voxel-based morphometry in normal aging. *Neuroimage* 48 371–380. 10.1016/j.neuroimage.2009.06.043 19559801 PMC2741580

[B35] Ito U. Kuroiwa T. Nagasao J. Kawakami E. Oyanagi K. (2006). Temporal profiles of axon terminals, synapses and spines in the ischemic penumbra of the cerebral cortex: ultrastructure of neuronal remodeling. *Stroke* 37 2134–2139. 10.1161/01.STR.0000231875.96714.b1 16809554

[B36] Johnson W. Mcgue M. Iacono W. G. (2005). Disruptive behavior and school grades: genetic and environmental relations in 11-year-olds. *Br. J. Educ. Psychol.* 97 391–405. 10.1037/0022-0663.97.3.391

[B37] Kuchinad A. Schweinhardt P. Seminowicz D. A. Wood P. B. Chizh B. A. Bushnell M. C. (2007). Accelerated brain gray matter loss in fibromyalgia patients: premature aging of the brain? *J. Neurosci.* 27 4004–4007. 10.1523/JNEUROSCI.0098-07.2007 17428976 PMC6672521

[B38] Li Y. Wang H. Niu L. Zhou Y. (2007). Chronic morphine exposure alters the dendritic morphology of pyramidal neurons in visual cortex of rats. *Neurosci. Lett.* 418 227–231. 10.1016/j.neulet.2007.03.023 17466454

[B39] Lin J. J. Dabbs K. Riley J. D. Jones J. E. Jackson D. C. Hsu D. A. (2014). Neurodevelopment in new-onset juvenile myoclonic epilepsy over the first 2 years. *Ann. Neurol.* 76 660–668. 10.1002/ana.24240 25087843 PMC4362677

[B40] Liu P. Yang J. Wang G. Liu Y. Liu X. Jin L. (2016). Altered regional cortical thickness and subcortical volume in women with primary dysmenorrhoea. *Eur. J. Pain* 20 512–520. 10.1002/ejp.753 26223337

[B41] Lydiard R. B. (2001). Irritable bowel syndrome, anxiety, and depression: what are the links? *J. Clin. Psychiatry* 62 38–45. 12108820

[B42] Mao C. P. Yang H. J. (2015). Smaller amygdala volumes in patients with chronic low back pain as compared to healthy control subjects. *J. Pain* 16 1366–1376. 10.1016/j.jpain.2015.08.012. 26431880

[B43] May A. (2008). Chronic pain may change the structure of the brain. *Pain* 137 7–15. 10.1016/j.pain.2008.02.034 18410991

[B44] Mayer E. A. Tillisch K. (2011). The brain-gut axis in abdominal pain syndromes. *Annu. Rev. Med.* 62 381–396. 10.1146/annurev-med-012309-103958 21090962 PMC3817711

[B45] Monuki E. S. Walsh C. A. (2001). Mechanisms of cerebral cortical patterning in mice and humans. *Nat. Neurosci.* 4(Suppl.) 1199–1206. 10.1038/nn770 11687830

[B46] Olson I. R. Plotzker A. Ezzyat Y. (2007). The enigmatic temporal pole: a review of findings on social and emotional processing. *Brain* 130 1718–1731. 10.1093/brain/awm052 17392317

[B47] Pochon J. B. Levy R. Fossati P. Lehericy S. Poline J. B. Pillon B. (2002). The neural system that bridges reward and cognition in humans: an fMRI study. *Proc. Natl. Acad. Sci. U.S.A.* 99 5669–5674. 10.1073/pnas.082111099 11960021 PMC122829

[B48] Rakic P. Ayoub A. E. Breunig J. J. Dominguez M. H. (2009). Decision by division: making cortical maps. *Trends Neurosci.* 32 291–301. 10.1016/j.tins.2009.01.007 19380167 PMC3601545

[B49] Raznahan A. Shaw P. Lalonde F. Stockman M. Wallace G. L. Greenstein D. (2011). How does your cortex grow? *J. Neurosci.* 31 7174–7177. 10.1523/JNEUROSCI.0054-11.2011 21562281 PMC3157294

[B50] Rodriguez-Raecke R. Niemeier A. Ihle K. Ruether W. May A. (2009). Brain gray matter decrease in chronic pain is the consequence and not the cause of pain. *J. Neurosci.* 29 13746–13750. 10.1523/JNEUROSCI.3687-09.2009 19889986 PMC6666725

[B51] Ruff R. M. Light R. H. Parker S. B. Levin H. S. (1996). Benton controlled oral word association test: reliability and updated norms. *Arch. Clin. Neuropsychol.* 11 329–338. 10.1016/0887-6177(95)00033-X 14588937

[B52] Ruff R. M. Light R. H. Parker S. B. Levin H. S. (1997). The psychological construct of word fluency. *Brain Lang.* 57 394–405. 10.1006/brln.1997.1755 9126423

[B53] Rypma B. D’Esposito M. (2000). Isolating the neural mechanisms of age-related changes in human working memory. *Nat. Neurosci.* 3 509–515. 10.1038/74889 10769393

[B54] Satsangi J. Silverberg M. S. Vermeire S. Colombel J. F. (2006). The Montreal classification of inflammatory bowel disease: controversies, consensus, and implications. *Gut* 55 749–753. 10.1136/gut.2005.082909 16698746 PMC1856208

[B55] Sauzéon H. Raboutet C. Rodrigues J. Langevin S. Schelstraete M.-A. Feyereisen P. (2011). Verbal knowledge as a compensation determinant of adult age differences in verbal fluency tasks over time. *J. Adult Dev.* 18 144–154. 10.1007/s10804-010-9107-6

[B56] Schmidt-Wilcke T. Leinisch E. Straube A. Kämpfe N. Draganski B. Diener H. (2005). Gray matter decrease in patients with chronic tension type headache. *Neurology* 65 1483–1486. 10.1212/01.wnl.0000183067.94400.80 16275843

[B57] Schwedt T. J. Berisha V. Chong C. D. (2015). Temporal lobe cortical thickness correlations differentiate the migraine brain from the healthy brain. *PLoS ONE* 10:e0116687 10.1371/journal.pone.0116687 PMC4332661 25679805

[B58] Segonne F. Dale A. M. Busa E. Glessner M. Salat D. Hahn H. K. (2004). A hybrid approach to the skull stripping problem in MRI. *Neuroimage* 22 1060–1075. 10.1016/j.neuroimage.2004.03.032 15219578

[B59] Seminowicz D. A. Labus J. S. Bueller J. A. Tillisch K. Naliboff B. D. Bushnell M. C. (2010). Regional gray matter density changes in brains of patients with irritable bowel syndrome. *Gastroenterology* 139 48–57. 10.1053/j.gastro.2010.03.049 20347816 PMC2902717

[B60] Sheehan T. J. Fifield J. Reisine S. Tennen H. (1995). The measurement structure of the center for epidemiologic studies depression scale. *J. Pers. Assess.* 64 507–521. 10.1207/s15327752jpa6403_9 7760258

[B61] Socha A. Cooper C. A. Mccord D. M. (2010). Confirmatory factor analysis of the M5-50: an implementation of the international personality item pool item set. *Psychol. Assess.* 22 43–49. 10.1037/a0017371 20230150

[B62] Sowell E. R. Peterson B. S. Thompson P. M. Welcome S. E. Henkenius A. L. Toga A. W. (2003). Mapping cortical change across the human life span. *Nat. Neurosci.* 6 309–315. 10.1038/nn1008 12548289

[B63] Sumerall S. W. Timmons P. L. James A. L. Ewing M. J. Oehlert M. E. (1997). Expanded norms for the controlled oral word association test. *J. Clin. Psychol.* 53 517–521. 9257231 10.1002/(sici)1097-4679(199708)53:5<517::aid-jclp14>3.0.co;2-h

[B64] Talairach J. Tournoux P. (1988). *Co-Planar Stereotaxic Atlas of the Human Brain: 3- Dimensional Proportional System – an Approach to Cerebral Imaging*. New York, NY: Thieme Medical Publishers.

[B65] Terry R. D. Deteresa R. Hansen L. A. (1987). Neocortical cell counts in normal human adult aging. *Ann. Neurol.* 21 530–539. 10.1002/ana.410210603 3606042

[B66] Wartolowska K. Hough M. G. Jenkinson M. Andersson J. Wordsworth B. P. Tracey I. (2012). Structural changes of the brain in rheumatoid arthritis. *Arthritis Rheum.* 64 371–379. 10.1002/art.33326 21905009

[B67] Watson D. (1988). Intraindividual and interindividual analyses of positive and negative affect – their relation to health complaints, perceived stress, and daily activities. *J. Pers. Soc. Psychol.* 54 1020–1030. 3397861 10.1037//0022-3514.54.6.1020

[B68] Watson D. Clark L. A. Carey G. (1988a). Positive and negative affectivity and their relation to anxiety and depressive-disorders. *J. Abnorm. Psychol.* 97 346–353. 10.1037/0021-843X.97.3.346 3192830

[B69] Watson D. Clark L. A. Tellegen A. (1988b). Development and validation of brief measures of positive and negative affect – the panas scales. *J. Pers. Soc. Psychol.* 54 1063–1070. 10.1037/0022-3514.54.6.1063 3397865

[B70] Wechsler D. (1997). *WAIS-III, Wechsler Adult Intelligence Scale: Administration and Scoring Manual*. San Antonio, TX: Psychological Corporation.

[B71] Wierenga L. M. Langen M. Oranje B. Durston S. (2014). Unique developmental trajectories of cortical thickness and surface area. *Neuroimage* 87 120–126. 10.1016/j.neuroimage.2013.11.010 24246495

[B72] Winkler A. M. Kochunov P. Blangero J. Almasy L. Zilles K. Fox P. T. (2010). Cortical thickness or grey matter volume? The importance of selecting the phenotype for imaging genetics studies. *Neuroimage* 53 1135–1146. 10.1016/j.neuroimage.2009.12.028 20006715 PMC2891595

