# Supplementary material for: Structural Imaging Changes and Behavioral Correlates in Patients with Crohn’s Disease in Remission
Source: Front Hum Neurosci. 2016 Sep 16;10:460. doi: 10.3389/fnhum.2016.00460 (PMC5025433; doi:10.3389/fnhum.2016.00460)
Supplement: Supplementary file 1 [file Presentation_1.PDF]

**Supplementary Table 1: Regions showing Cortical Thickness differences between Patients and Controls**					
t value	Extent of Area (mm2)	Talairach Coordinates			Cerebral region
		X	Y	Z	
****3.34**	1437.51	-12	52	0.2	Left superior frontal
*******corrected for multiple comparisons, clusterwise p =0 .011*					
***Left hemisphere (patients>controls)**					
3.3943	901.25	-42	-84	-1	lateral occipital
3.3206	1261.79	-12	53	2	superior frontal
3.2299	314.06	-47	-21	39	post central
2.8797	218.58	-35	32	13	rostral middle frontal
2.2828	190.83	-62	-16	18	post central
2.2173	302.28	-49	-28	1	superior temporal
1.8548	203.76	-11	-12	49	superior frontal
1.7585	162.75	-55	-54	-17	inferior temporal
1.665	132.9	-38	51	5	rostral middle frontal
1.639	101.31	-13	29	-18	lateral orbito frontal
1.5101	41.69	-20	-54	5	isthmus cingulate
1.4436	28.92	-48	-27	55	post central
1.3459	17.16	-23	-83	29	superior parietal
***Left hemisphere (patients < controls)**					
-4.892	1072.91	-43.2	32.9	-9.7	pars triangularis
-4.6805	257.93	-47	3.4	7	precentral

-4.0131	552.65	-10	-55.8	57	precuneus
-2.4076	659.92	-4.8	-74.3	4.2	lingual
-2.0676	166.77	-34.4	5.6	1.4	insula
-1.987	261.91	-4.1	-32.8	69	precentral
-1.7735	95.09	-46	11.1	-22.7	superior temporal
-1.7202	91.3	-51.7	-0.7	34.3	precentral
-1.7063	62.5	-23.8	-23	62.7	precentral
-1.6093	220.85	-41.8	16.7	38.7	caudal middle frontal
-1.3816	67.85	-8	-71.8	11.4	pericalcarine
-1.3712	20.49	-51.3	28.8	3.6	pars triangularis
-1.3073	2.13	-31.8	10.6	50.3	caudal middle frontal
-1.3069	1.88	-43.4	-10.3	-39.2	inferior temporal
***Right hemisphere (patients>controls)**					
4.05	315.41	57	-59	5	middle temporal
2.62	405.37	58	-14	36	post central
2.36	481.46	46	-80	6	lateral occipital
1.92	99.88	48	-56	19	inferior parietal
1.86	523.84	22	57	1	rostral middle frontal
1.85	68.43	54	4	18	precentral
1.62	36.60	10	26	37	superior frontal
1.61	122.28	31	39	-9	lateral orbitofrontal
1.61	41.93	34	-11	11	insula
1.57	57.27	6	52	-19	medial orbitofrontal
1.42	26.4	42	-28	46	post central
1.39	21.26	41	-63	-11	fusiform
1.33	4.36	56	-43	26	supramarginal
***Right hemisphere (patients < controls)**					

-3.16	178.04	20	24	46	superior frontal
-2.24	430.73	41	3	49	caudal middle frontal
-2.04	152.66	11	11	52	superior frontal
-1.97	104.73	22	-2	56	superior frontal
-1.92	74.68	18	41	38	superior frontal
-1.89	68.85	24	-57	64	superior parietal
-1.72	88.21	60	-13	-3.1	superior temporal
-1.48	24.01	9	-54	53	precuneus
-1.37	45.24	5	-72	14	pericalcarine

*****_uncorrected 0.01<p<0.00001;_

Supplementary Table 2: Regions showing Cortical Surface Area differences between Patients and Controls					
t value	Extent of Area (mm2)	Talairach Coordinates			Cerebral region
		X	Y	Z	
***Left Hemisphere (controls > patients)**					
2.35	237.30	12	39	42	precuneus
2.02	384.73	-41	11	7	pars opercularis
1.89	534.21	-31	-53	-12	fusiform
1.79	112.41	-9	-33	52	paracentral
1.77	158.81	-53	-12	-10	superior temporal
1.76	217.51	-26	-4	44	caudal middle frontal
1.73	70.41	-37	5	23	pars opercularis
1.68	74.81	-36	-28	3	insula
1.66	192.52	-36	-33	21	supramarginal
1.49	67.17	-62	-31	1	superior temporal
1.43	80.44	-10	-30	66	precentral
1.34	5.99	-38	-37	37	supramarginal
***Left Hemisphere (patients > controls)**					
-2.28	49.22	-31	4	-31	temporal pole
-2.19	268.61	-11	64	14	superior frontal
-1.94	431.41	-33	42	19	rostral middle frontal
-1.60	16.16	-35	2	-18	insula
-1.44	67.92	-9	-79	3	pericalcarine
-1.34	28.64	-46	-31	-23	inferior temporal

***Right Hemisphere (controls > patients)**					
2.54	512.30	33	3	51	caudal middle frontal
2.35	177.20	48	-0.1	-17.5	superior temporal
1.96	226.11	33	12	3	insula
1.91	174.33	17	-28	41	paracentral
1.91	147.08	3	23	16	caudal anterior cingulate
1.49	48.37	7	-70	48	precuneus
1.40	52.03	49	-29	27	supramarginal
1.38	29.14	62	-40	-9	middle temporal
1.31	4.81	42	26	16	rostral middle frontal
***Right Hemisphere (patients > controls)**					
-2.47	345.95	32	6	-32	temporal pole
-2.02	414.89	19	11	61	superior frontal
-1.88	497.62	7	42	43	superior frontal
-1.87	96.55	5	-56	15	precuneus
-1.51	90.02	22	28	35	superior frontal
-1.51	40.13	56	-2	36	precentral
-1.49	64.91	16	16	-21	lateral orbitofrontal
-1.33	13.52	18	-35	68	post central
***uncorrected** *0.01<p<0.00001;*					

Supplementary Table 3A

Significant correlations: Sub-cortical volumes and behavioral measures for patients

PATIENTS (N=19)	Left Putamen	Left Pallidum	Left Hippocampus	Left Amygdala	Left Caudate	Right Thalamus	Right Putamen	Right Pallidum	Right Caudate
**Disease duration**				**-0.48** (.04)					
**Digit Span Backward**					**.47** (.05)				**.52** (.03)
**BIS**			**.46** (.05)	**.48** (.04)					
**Extraversion**		**+0.45** (.05)	**.46** (.05)						
**Agreeableness**		**.61** (.006)			**.54** (.01)		**.46** (.05)	**.60** (.008)	**.48** (.04)
**Conscientiousness**			**.46 (.05)**	**+0.48** (0.04)					
**Emotional Stability**						**.48** (.04)			
**Openness**	**.60** (.008)						**.48** (.04)	**.68** (.002)	

Note: ***Correlation values are shown in bold, and significance values in parentheses.***

Supplementary Table 3B

Significant correlations: Sub-cortical volumes and behavioral measures for healthy controls

CONTROLS (N=20)	Left Thalamus	Left Putamen	Left Pallidum	Left Amygdala	Left Caudate	Right Putamen	Right Pallidum	Right Hippocampus	Right Amygdala	Right Caudate
**Digit Span Forward**			**-.54** (.01)		**-.53** (.02)			**- .48** (.03)	**-.57** (.01)	**-.48** (.03)
**Digit Span Backward**									**-.42** (.06)	
**BAS Drive**			**.49** (.03)		**.60** (.007)	**.45** (.05)	**.45** (.05)			
**BAS Fun seeking**					**.46** (.04)					**.44** (.05)
**Positive Affect**					**-.44** (.05)	**-.51** (.02)				**-.46** (.04)
**Negative Affect**	**-.45** (.05)							**-.44** (.05)		
**Agreeableness**				**.63** (.004)						
**Conscientious ness**				**-.49** (.03)						
**Openness**		**-.57** (.01)					**-.49** (.03)			
**Depression**		**.48** (.03)			**.60** (.006)	**.55** (.01)				

Note: *Correlation values are shown in bold, and significance values in parentheses.*
